# A rare case of a sporadic retroperitoneal hemangioblastoma

**DOI:** 10.1093/jscr/rjad629

**Published:** 2023-11-20

**Authors:** Maida Malagic Polutak, Mark Hartel

**Affiliations:** University Department of Medicine, Division of General Internal and Emergency Medicine, Cantonal Hospital Aarau, Aarau CH-5001, Switzerland; Department of General and Visceral Surgery, Cantonal Hospital Aarau, Aarau CH-5001, Switzerland

**Keywords:** hemangioblastoma, sporadic hemangioblastoma, extraneural hemangioblastoma, retroperitoneal tumors

## Abstract

Hemangioblastoma is a rare, benign, and morphologically distinctive tumor. In most cases, the tumor involves the central nervous system. Extraneural occurrences are rare, with just a few reports of hemangioblastoma situated outside of neural tissue, such as the retroperitoneum. We report a case of sporadic retroperitoneal hemangioblastoma in an 87-year-old male patient, diagnosed as an incidental finding in a CT scan performed because of kidney stone disease. The CT scan showed a mass in the retroperitoneum posterior to the inferior vena cava. The patient reported no remarkable symptoms. We describe our path to diagnosis, the possible differential diagnosis for retroperitoneal masses, and the histopathologic features of the tumor. There are <250 reported extra neuraxial hemangioblastomas and just 14 reported cases situated outside of the neural tissue. Our case is the eighth case report of a hemangioblastoma arising from the soft tissue of the retroperitoneum.

## Introduction

Hemangioblastoma is a rare, benign, and highly vascular neoplasm of uncertain histogenesis, consisting of networks of small blood vessels interspersed with lipid-laden stromal cells [1]. This tumor can occur sporadically or as a part of von Hippel–Lindau-Syndrome, a syndrome manifested by various benign and malignant tumors. About 75% of hemangioblastomas occur sporadically, while the remaining 25% occur in the setting of von Hippel-Lindau-Syndrome. Sporadic hemangioblastomas tend to occur in adults, whereas von Hippel-Lindau-Sydrome-associated ones tend to affect younger patients [2]. Hemangioblastomas typically occur in neural tissues in the brain, spinal cord, and retina. Extraneural hemangioblastoma is rare, with just a small series of case reports about this condition. Brodkey et al. published in 1995 the first case of hemangioblastoma develops in the soft tissues, arising in the radial nerve [3]. Other reported extraneural localizations are in the peripheral nerves, liver, lung, pancreas, retroperitoneum, kidney, urinary bladder, popliteal fossa, and nasal skin. They can present with various symptoms, depending on their size and location. In this case report, we will provide a detailed overview of a sporadic retroperitoneal hemangioblastoma in an 87-year-old male patient.

## Case report

**Figure 1:**
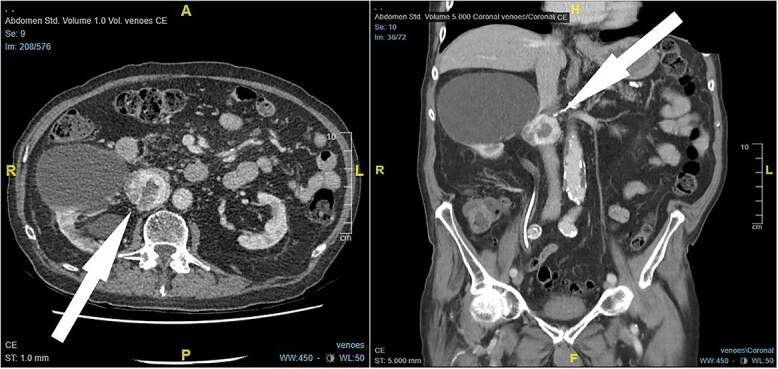
CT scan findings, 4.5 × 4 × 4 cm right retroperitoneal mass posterior to the inferior vena cava, with compression on the inferior vena cava and the right renal artery (arrow).

**Figure 2:**
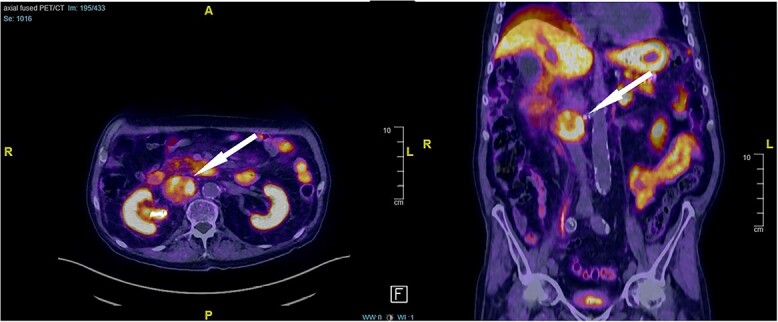
DOTATE-PET/CT: sizeable retrocaval mass with edge-accentuated radionuclide storage, compatible with a paraganglioma or a lymph node metastasis of a neuroendocrine tumor (arrow).

An 87-year-old male Caucasian patient with a history of cardiac co-morbidities, diabetes mellitus type 2, and chronic kidney disease stage 4 was referred to our hospital for clinical evaluation and a non-contrast abdominal–pelvic CT scan because of right side pain with a history of kidney stone disease, with recurrent ureteral stones. The CT scan showed a small distal ureteral stone on the right side without the need for surgical intervention. Incidentally, he had a retroperitoneal mass (4.4 × 4 × 4 cm) posterior to the inferior vena cava, with compression on the inferior vena cava and the right renal artery (Fig. 1). Our radiologist classified this unexpected incidental finding as a possible neuroendocrine tumor, with the recommendation to perform a DOTATE-PET/CT scan. The DOTATE-PET/CT showed a sizeable retrocaval mass with edge-accentuated radionuclide storage, compatible with a paraganglioma or a lymph node metastasis of a neuroendocrine tumor (Fig. 2). The ultimate etiology remained unclear, despite the radiological scans. Our blood tests showed a normal level of hormones in the blood and urine, excluding hormonally active tumors. The only noticeable value in the blood was the elevated creatinine as a part of the chronic kidney disease. One of our assumptions was that the tumor compression on the renal artery impacts the patient’s kidney function. The patient reported no remarkable symptoms.

Because of the compression on the right renal artery and the unclear diagnosis, we decided to perform surgery. The resection of the tumor was performed through an open transperitoneal approach with a midline incision. Intraoperatively, the mass was adherent to the inferior vena cava and the right renal artery, making the separation very difficult. Ultimately, we removed the tumor *in toto*, which showed a pseudo encapsulation and an uneven surface. The patient recovered without complications and was discharged home on Day 6 after surgery. A few weeks after the surgery, the kidney function improved slightly.

Our pathologists found a circumscribed tumor surrounded by a connective tissue capsule, consisting of numerous thin-walled vessels of small to medium diameter, lined by flat endothelial cells without significant atypia, and on the other hand, epithelioid cells between the epithelioid cells with round oval nuclei, bright chromatin, partly with nuclear pseudo-inclusions, and pale-eosinophilic cytoplasm, without recognizable cell borders. Some of these epithelioid cells show a vacuolated cytoplasm. No mitotic figures are seen, no necrosis. The endothelia of the blood vessels are stained by immunohistochemistry using CD31, CD34, and ERG staining, but no interstitial cells are stored between the vessels. Interstitial epithelioid cells showed diffuse S100- and focally inhibin-positive. There was no expression of D2-40, PAX8, AE1/AE3, CK8/18, RCC, or CD10. Ki67 proliferation index was ~1–2%.

## Discussion

Hemangioblastomas are rare tumors that typically occur in the central nervous system, in 25% of the cases as part of von Hippel-Lindau disease [4]. Occasionally they manifest in other locations, such as the retroperitoneal space, like in our reported case. Our literature search showed <250 reported extra neuraxial hemangioblastomas, most occurring in para neuraxial structures [5–12]. We found 14 reported cases of sporadic hemangioblastoma situated outside of the neural tissue, including our case, which is the eighth case report of a hemangioblastoma arising from the retroperitoneal soft tissue (Table 1). None of the reported retroperitoneal hemangioblastomas was von Hippel-Lindau-Syndrome associated. Because of the rare occurrence in extraneural sites, hemangioblastomas are usually not considered in the differential diagnosis. In addition, they morphologically can mimic many tumor types, making the diagnosis more difficult.

**Table 1:** Overview of the case reports about retroperitoneal hemangioblastomas

Autor/Year	Name	Sample size	Gender	Age
Lu *et al.* 2023	Hemangioblastoma arising from the retroperitoneum: a case report	1	Female	51
Jalikis *et al.* 2017	Sporadic retroperitoneal hemangioblastoma: report of a case and review of the literature	1	Male	79
Huang *et al.* 2014	Sporadic hemangioblastoma of the retroperitoneum	1	Male	59
Yoshida *et al.* 2010	Soft-tissue hemangioblastoma of the retroperitoneum: a case study and review of the literature	1	Female	71
Fanburg-Smith *et al.* 2000	Retroperitoneal peripheral hemangioblastoma: a case report and review of the literature	1	Male	47
Nonaka *et al.* 2007	Extraneural hemangioblastoma: a report of five cases	2	Male Female	34 91

On the other hand, primary retroperitoneal tumors represent a very heterogeneous group of neoplastic and non-neoplastic entities that originate in the retroperitoneal tissues without arising from the organs located there. We can distinguish here between solid and cystic tumors and between neoplastic and non-neoplastic tumors. The best examples therefore are lymphoid tumors, sarcomas, neurogenic tumors, mature teratomas, Mullerian cysts, pancreatic pseudocysts, etc. [13–15]. They often share common imaging features, making the diagnosis even more difficult. Hemangioblastoma is generally present on imaging as sharply demarcated homogeneous masses composed of a cyst with non-enhancing walls, a mural nodule that vividly enhances, often with prominent serpentine flow voids.

If the diagnosis remains unclear, surgery should be the next step. The decision for laparoscopic or open surgery depends mainly on the location, the contact with the adjacent structures, and the surgeon’s experience. However, retroperitoneal masses are challenging to reach, so we prefer the open approach in our clinic.

In the histopathology, the characteristics of hemangioblastoma included circumscribed borders, often solid, lack of mitotic figures, fine vacuoles and intracytoplasmic lipids, alternation of cellular and paracellular areas, nuclei pleomorphism, and arborizing capillary-sized vessels [16]. Immunohistochemical studies performed on hemangioblastomas showed three markers that are constantly expressed in the tumor, which are vimentin, S-100 protein, and neuron-specific enolase. A frequent expression of Inhibin was also reported. All other analyzed markers, such as cytokeratins, MSA, desmin, calponin, CD56, glial fibrillary acidic protein, factor VIII, and *Ulex europaeus* I, have demonstrated a very variable expression [2].

## Conclusion

Our work shows the wide range of possible differential diagnoses for retroperitoneal masses. A hemangioblastoma arising from the soft tissue of the retroperitoneum is very rare but should be considered in the differential diagnosis of unclear retroperitoneal masses. Due to the unspecific symptoms and imaging features, clinical and imaging diagnostics are not enough, but a pathohistological examination is always required in such cases.
